# Esc peptides as novel potentiators of defective cystic fibrosis transmembrane conductance regulator: an unprecedented property of antimicrobial peptides

**DOI:** 10.1007/s00018-021-04030-2

**Published:** 2021-12-31

**Authors:** Loretta Ferrera, Floriana Cappiello, Maria Rosa Loffredo, Elena Puglisi, Bruno Casciaro, Bruno Botta, Luis J. V. Galietta, Mattia Mori, Maria Luisa Mangoni

**Affiliations:** 1 U.O.C. Genetica Medica, Istituto Di Ricovero E Cura a Carattere Scientifico (IRCCS) Istituto Giannina Gaslini, 16147 Genoa, Italy; 2 Department of Biochemical Sciences, Laboratory affiliated to Istituto Pasteur Italia-Fondazione Cenci Bolognetti, Sapienza University of Rome, 00185 Rome, Italy; 3 Center for Life Nano- and Neuro-Science, Fondazione Istituto Italiano Di Tecnologia (IIT), 00161 Rome, Italy; 4 Department of Chemistry and Technology of Drugs, “Department of Excellence 2018−2022”, Sapienza University, P. le Aldo Moro 5, 00185 Rome, Italy; 5 Telethon Institute of Genetics and Medicine (TIGEM), 80078 Pozzuoli, NA Italy; 6 Department of Translational Medical Sciences, University of Napoli “Federico II”, 80131 Napoli, Italy; 7 Department of Biotechnology, Chemistry and Pharmacy, Department of Excellence 2018−2022, University of Siena, 53100 Siena, Italy

**Keywords:** Antimicrobial peptides, Innate immunity, CFTR potentiators, Antibiotic-resistance, Lung pathology, Pulmonary drug delivery

## Abstract

Mutations in the cystic fibrosis (CF) transmembrane conductance regulator (CFTR) protein lead to persistent lung bacterial infections, mainly due to *Pseudomonas aeruginosa,* causing loss of respiratory function and finally death of people affected by CF. Unfortunately, even in the era of CFTR modulation therapies, management of pulmonary infections in CF remains highly challenging especially for patients with advanced stages of lung disease. Recently, we identified antimicrobial peptides (AMPs), namely Esc peptides, with potent antipseudomonal activity. In this study, by means of electrophysiological techniques and computational studies we discovered their ability to increase the CFTR-controlled ion currents, by direct interaction with the F508del-CFTR mutant. Remarkably, this property was not explored previously with any AMPs or peptides in general. More interestingly, in contrast with clinically used CFTR modulators, Esc peptides would give particular benefit to CF patients by combining their capability to eradicate lung infections and to act as promoters of airway wound repair with their ability to ameliorate the activity of the channel with conductance defects. Overall, our findings not only highlighted Esc peptides as the first characterized AMPs with a novel property, that is the potentiator activity of CFTR, but also paved the avenue to investigate the functions of AMPs and/or other peptide molecules, for a new up-and-coming pharmacological approach to address CF lung disease.

**Supplementary Information:**

The online version contains supplementary material available at 10.1007/s00018-021-04030-2.

## Introduction

Cystic Fibrosis (CF) is the most common autosomal recessive disease among individuals of caucasian origin, with an incidence of approximately 1 out of 2,500 new births in Europe and 1 out of 3,500 newborns in the United States of America (USA) [1–3]. There are over 350 different mutations (http://www.genet.sickkids.on.ca) affecting transcription, synthesis, trafficking, turnover, or function of the CF transmembrane conductance regulator (CFTR) protein which is an anion-selective channel controlling chloride and bicarbonate transport mainly at the apical plasma membrane of secretory epithelia of different organs, including the lungs and the intestine [4, 5]. CFTR bears two membrane spanning domains (MSD) that form a pore; two cytoplasmic nucleotide-binding regions (NBD1 and NBD2) that bind and hydrolyze ATP, and the regulatory R domain [6]. Phosphorylation at multiple sites in the R domain by cAMP-dependent protein kinase A (PKA) [7], as well as interaction with ATP at the two NBDs, control channel opening [8, 9]. Inversely, ATP hydrolysis at NBD2 drives to channel closing [10]. The most prevalent CF-associated mutation is the loss of phenylalanine at position 508 (F508del-CFTR) which causes an incorrectly folded protein that is rapidly degraded by the ubiquitin–proteasome pathway [11]. Because of this trafficking fault, a small fraction of F508del-CFTR reaches the plasma membrane [12]; however, the mutated protein also exhibits a defect in channel gating [13]. Consequently, the flow of anions (chloride and bicarbonate) outside the cells is decreased thus leading to airway surface dehydration. In the respiratory tract, the formation of a sticky and dehydrated mucus occurs on the airway surfaces with serious decay of mucociliary defense system. This favors the entrapment and accumulation of inhaled microbes, such as *Pseudomonas aeruginosa* which quickly gives rise to biofilm communities [14]. Biofilms are complex aggregates of bacteria encased in a self-generated matrix of extracellular polymeric substances that escape antibiotic treatments and host immune response [15]. Hence, a chronic pulmonary infection takes hold with progressive damage to the pulmonary tissue.

With the aim to search for appropriate pharmacological approaches to correct the dysfunction of mutant CFTR, CF research community has been focusing its attention on small molecules able (i) to promote the delivery of the defective channel to the plasma membrane (namely correctors) by direct binding to CFTR or to other proteins involved in the protein trafficking machinery, and/or (ii) to improve ions permeation through CFTR (namely potentiators) by binding to NBDs or MSDs. However, only a few compounds have progressed to clinical trials [16]. The correctors lumacaftor (VX-809), tezacaftor (VX-661), elaxacaftor (VX-445) and the potentiator ivacaftor (VX-770) are CFTR modulators currently used in the therapy of CF [17–19]. Since their advent, the clinical course of patients has improved dramatically. Both treatments based on ivacaftor (trade name Kalydeco) for "gating" mutations and the triple combination elexacaftor/tezacaftor/ivacaftor (trade name Trikafta) for F508del mutation in double or single copy are effective for patients, allowing them to finally come out from the lung transplant list, in many cases. However, despite the remarkable effectiveness in restoring the channel’s functions, CFTR modulators are unable to eradicate bacterial infections, especially in CF patients with irreversible lesions of the lung structure and/or bronchiectasis [20].

During the last years, several studies have been conducted to identify compounds with suitable antibacterial properties. Antimicrobial peptides (AMPs) are a promising and effective therapeutic resource [21, 22]. Indeed, they rapidly kill microbial pathogens by perturbing their plasma-membrane thus limiting the induction of bacterial resistance compared to conventional antibiotics [23]. In our laboratory, we identified a frog-skin derived membrane-active cationic AMP, i.e., Esc(1–21) GIFSKLAGKKIKNLLISGLKG-NH_2_ [24], with biocidal activity against the planktonic and the biofilm forms of *P. aeruginosa* strains at concentrations ranging from 1 to 25 µM [25]. By changing the configuration of two l-amino acids in the sequence of Esc(1–21), i.e., Leu^14^ and Ser^17^ with the corresponding d-enantiomers, we also demonstrated that the resulting diastereomer Esc(1–21)-1c is less susceptible to proteolytic degradation and more efficient in promoting re-epithelialization in monolayers of bronchial epithelial cells expressing either a functional or a mutated copy of CFTR (wt-CFBE and F508del-CFBE, respectively) [26]. Importantly, this is expected to accelerate healing of injured infected lung tissue by restoring its integrity. Furthermore, an earlier work carried out with fluorescent-labelled Esc(1–21)/Esc(1–21)-1c (Esc peptides) revealed their ability to penetrate both wt-CFBE and F508del-CFBE [26], with Esc(1–21) being mainly distributed at the perinuclear region, as reported also for the cationic human AMP LL-37 in A549 lung epithelial cells [27]. In addition, Esc(1–21)-1c has shown a higher effectiveness than the all-l counterpart in contrasting *P. aeruginosa* pulmonary infection in mice, being comparable in efficacy to colistin, the last-resort drug against life-threatening infections [28, 29], without eliciting toxicity or inflammatory side events [26, 28, 30].

With the aim to develop Esc peptides or derivatives as novel multifunctional drug candidates for ideal treatment of CF pulmonary disease, here we investigated their ability to affect ion currents controlled by CFTR by combining different electrophysiological techniques and computational studies. Considerably, we report on an unprecedented property of AMPs, that is the ability to act as potentiators of mutated CFTR by direct interaction with the defective protein.

## Results

### Esc peptides do not affect epithelial permeability

**Fig. 1:**
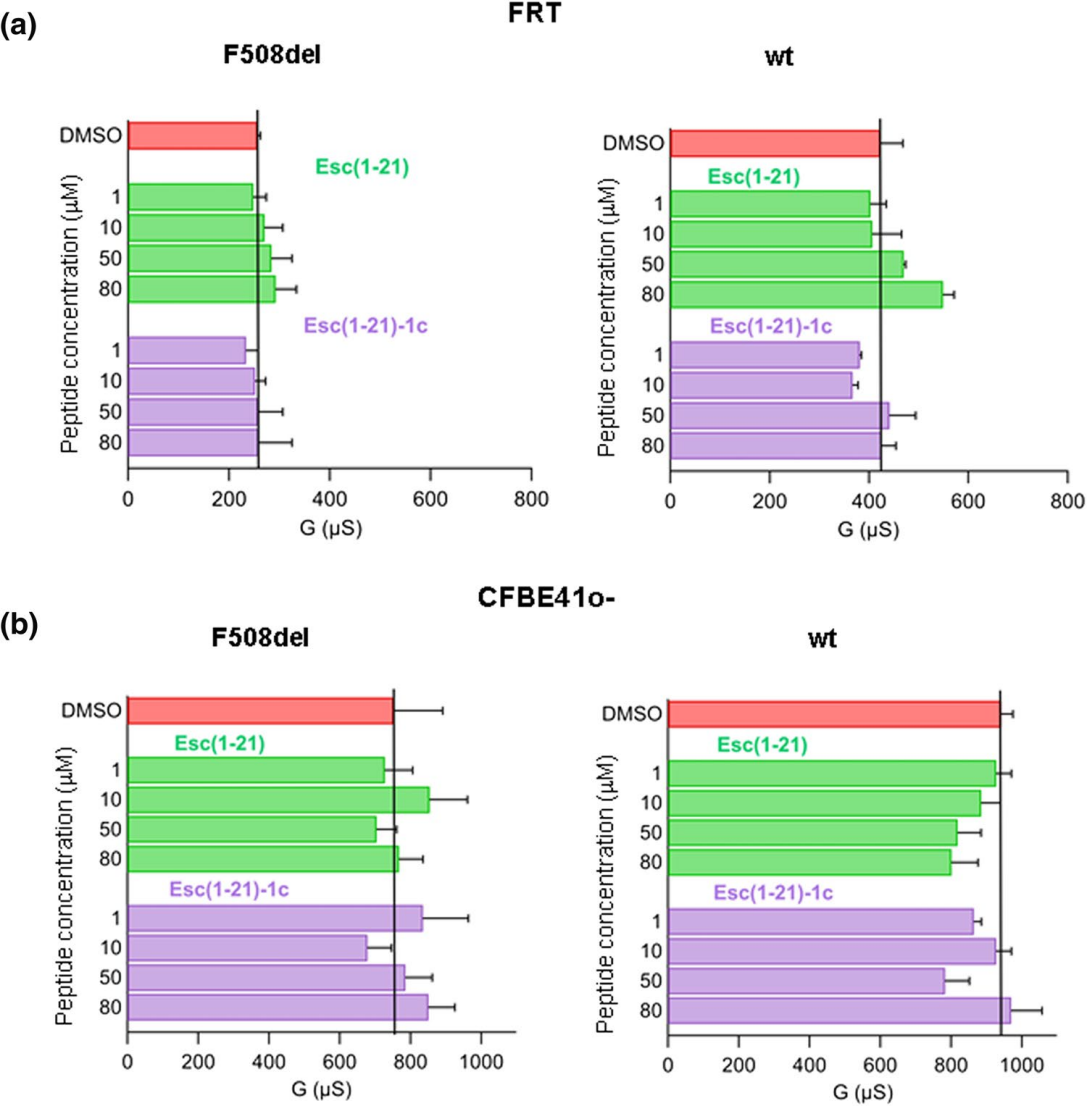
Effect of peptides on the epithelial integrity by the TEER assay on FRT (**a**) and CFBE41o-cells (**b**) expressing either F508del-CFTR (left panels) or wt-CFTR (right panels). Cells were treated for 24 h with the indicated peptides at different concentrations; afterwards, transepithelial conductance was measured and compared to that of DMSO-treated cells highlighted by the solid black line. Data are expressed as mean ± SEM from at least six independent experiments, each in triplicate. No significant differences were found among samples

The epithelial barrier integrity, that is vital for respiratory functions especially in CF lung, can be assessed by measuring the transepithelial electrical resistance (TEER) across a proper cellular monolayer mimicking the airway epithelium [31]. We initially monitored the TEER in polarized epithelia formed by Fischer rat thyroid (FRT) cells with stable expression of mutated or wild-type CFTR (F508del-FRT and wt-FRT, respectively), 24 h after peptide treatment at different concentrations. As for airway epithelial cells, FRT cells (which have been extensively used for studies of CFTR protein [32, 33]), form a cellular monolayer stabilized via intercellular junctions that regulate diffusion of water and solutes allowing the establishment of selectively permeable barriers [34]. Minor changes in TEER, reported as transepithelial electrical conductance (G) in Fig. 1a, were attained in comparison to the vehicle dimethyl sulfoxide (DMSO)-treated control samples, indicating that none of the two peptides can alter the epithelial entirety. Similar results were obtained for the bronchial epithelial cell line CFBE41o- expressing either F508del- or wt-CFTR (F508del-CFBE41o- or wt-CFBE41o-, Fig. 1b). The different G value between the two cell lines likely depends on both their intrinsic characteristics in the differentiation process of the epithelium [31, 35], and their ability to form tight junctions, as well as on the conditions used for cell culture.

### Esc peptides potentiate the activity of F508del-CFTR

To find out whether Esc peptides are able to improve the ion permeability of F508del-CFTR in FRT cell line, we tested these compounds in combination with forskolin (FSK), that is an activator of adenylate cyclase which in turn increases the intracellular cAMP level [36] required for CFTR activation. We compared the results of channel activity determined by FSK together with Esc peptides, to those obtained by the application of FSK alone or FSK plus genistein (GEN). GEN is a known potentiator of CFTR that accelerates channel opening and slows channel closure by direct interaction with the channel [37, 38].

**Fig. 2:**
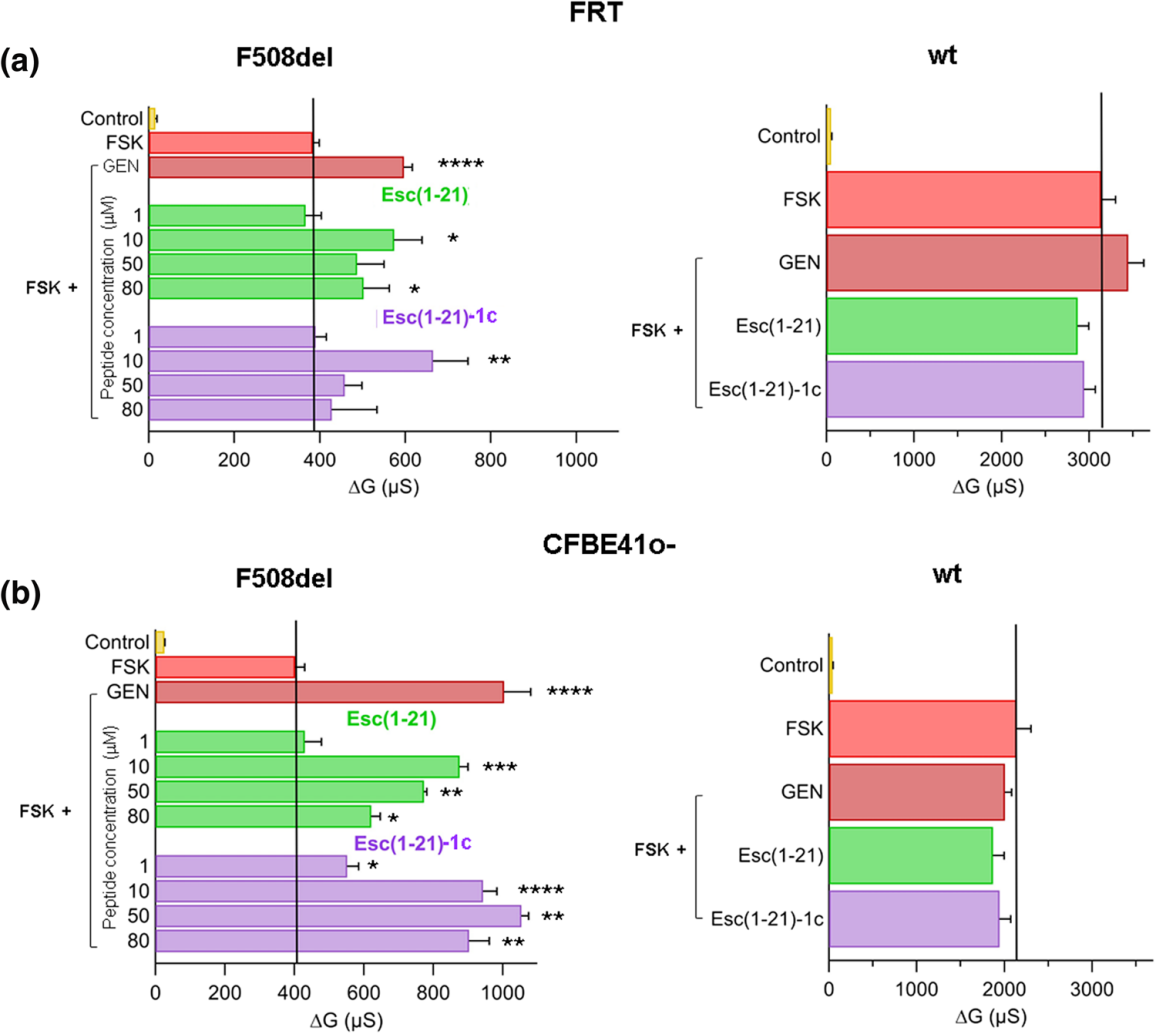
Potentiator effect of Esc peptides on CFTR activity. **a** and **b**, left panels F508del-FRT and F508del-CFBE41o- epithelia respectively were incubated for 24 h with 1 µM VX-809 (to allow the mutated protein to reach the apical membrane). CFTR-mediated transepithelial conductance was evaluated as the difference between the conductance measured after 10 min addition of 20 µM FSK alone or after addition of the combination FSK + 10 µM GEN or + Esc peptides (at different concentrations), and the conductance measured after CFTR-inhibition (delta conductance, ΔG). Cells pre-incubated with VX-809 but not activated were used as control. **a** and **b**, right panels FRT and CFBE41o- expressing wt-CFTR respectively were also treated with 20 µM FSK alone or with the combination FSK + 10 µM Esc peptides/GEN, as described above. All data are expressed as mean ± SEM from n ≥ 6 independent experiments. The level of statistical significance of samples *versus* FSK is indicated as follows: *, *p* < 0.05; **p < 0.01; ***p < 0.001; ****p < 0.0001. Comparison between data was done by Student’s t test. The solid black line highlights the delta conductance measured in epithelia treated with 20 µM FSK alone

The CFTR activity was evaluated by TEER as the difference between the transepithelial conductance measured after 10-min treatment of the epithelium with FSK alone or with the combination FSK plus GEN or plus Esc peptides (at different concentrations ranging from 1 to 80 µM) and the conductance measured after CFTR inhibition, due to the addition of the CFTR inhibitor PPQ102. Therefore, CFTR activity is indicated as delta conductance (ΔG). Interestingly, as shown in Fig. 2a (left panel), compared to FSK-treated samples, about 1.5-fold higher CFTR-mediated conductance was recorded when the epithelium was exposed to each Esc peptide at the concentration of 10 µM which was subsequently chosen for further experiments. This enhancement was slightly more pronounced for the Esc(1–21)-1c isomer carrying d-Leu^14^ and d-Ser^17^ and was comparable to that provoked by the potentiator GEN when combined, at the same concentration of 10 µM, with FSK. Similar data were also achieved for F508del-CFBE41o- (Fig. 2b left panel). As already found for other known potentiators such as GEN, the activity of the peptides on the mutated channel depended on their concentration. They gave a bell-shaped dose–response behaviour with a stimulatory effect at the lower concentrations and an inhibitory effect at the higher dosages [39]. Note that both F508del-FRT and F508del-CFBE41o- epithelia were pre-incubated for 24 h with 1 µM VX-809 to allow the mutated protein to reach the apical membrane.

In comparison, no increase in CFTR-mediated transepithelial conductance was noted in FRT and CFBE41o- cells expressing wild-type CFTR, upon treatment with FSK plus 10 µM of Esc peptides compared to FSK-treated samples (Fig. 2a and b, right panels) suggesting that CFTR phosphorylation triggered by FSK is sufficient to determine the complete channel activation.

### The potentiator effect of Esc peptides is preserved in primary airway epithelial cells

**Fig. 3:**
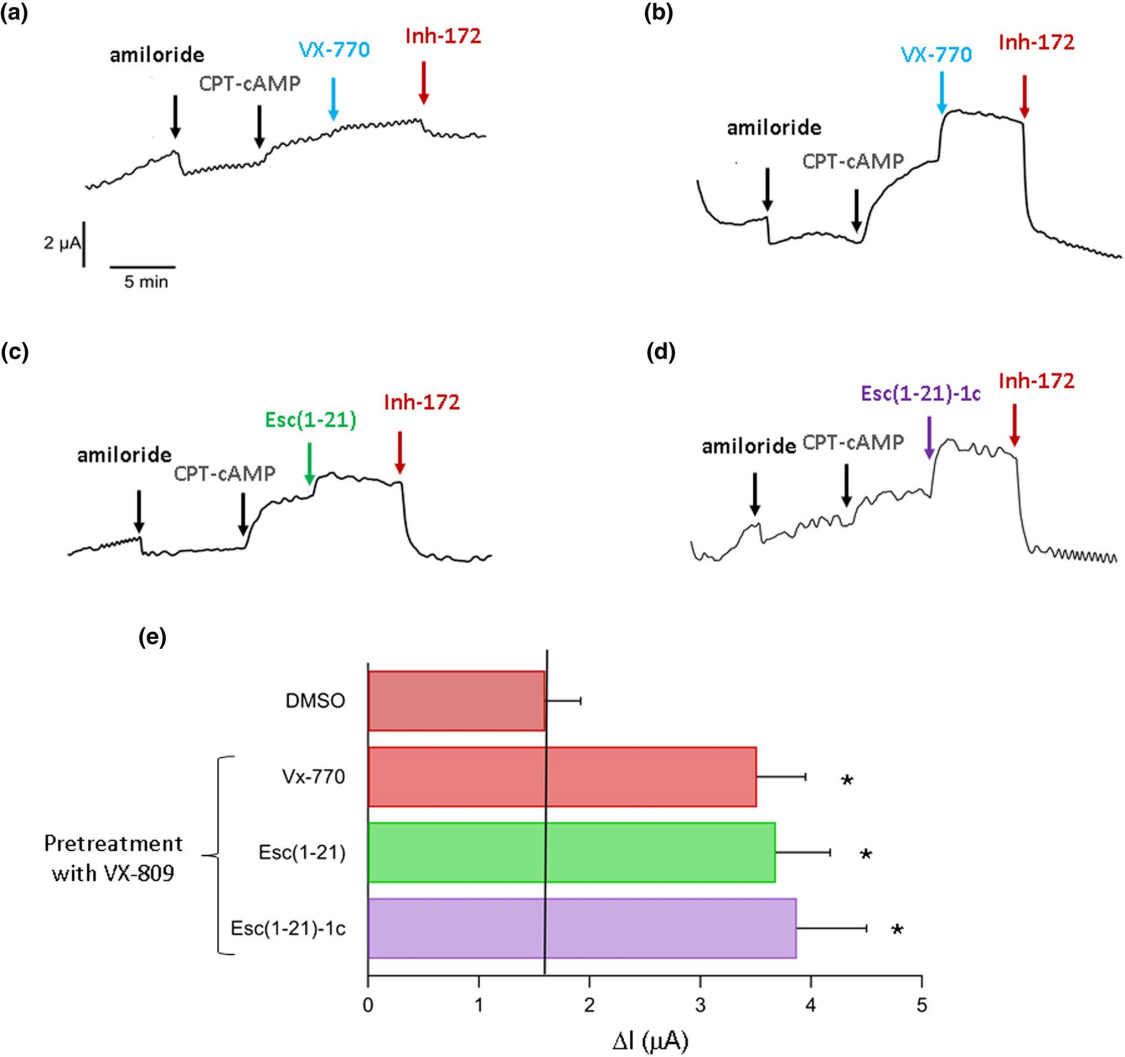
Effect of peptides on F508del-CFTR activity in primary airway epithelial cells by the short-circuit current technique. **a**–**d** Representative traces of currents recorded in a Ussing chamber from bronchial epithelial cells (HBE) derived from a homozygous F508del patient after 24 h incubation with DMSO (**a**) or with 1 μM VX-809 (**b**–**d**). The application of amiloride (10 µM) and CPT-cAMP (100 µM) is indicated over each record. The potentiator VX-770 (1 µM) was applied to untreated (**a**) and to corrector-treated cells (**b**), while Esc peptides at 10 µM were assayed in corrector-treated cells (**c** and **d**). **e** Bar graphs summarizing CFTR-mediated current from Ussing chamber recordings of HBE. This was calculated as the difference between the current amplitude measured after treatment of VX-809-preincubated cells with 1 µM VX-770 or with 10 µM Esc peptides and the current amplitude measured after CFTR inhibition (delta current, ΔI). The ΔI of DMSO-preincubated cells and subsequently exposed to VX-770 was also included for comparison. Data are the mean ± SEM from n ≥ 3 independent experiments. The level of statistical significance *versus* DMSO-treated samples is indicated as follows: *p < 0.05. Comparison between data was done using Student’s t test. The solid black line indicates the delta current measured in epithelia treated with DMSO

To validate the results obtained with both FRT and CFBE41o- cell lines, Esc peptides were further analyzed at 10 µM on primary homozygous F508del bronchial epithelial cells. Measurement of chloride transport by short-circuit current analysis was performed on the epithelia mounted in a perfusion chamber (Ussing Chamber). After blocking epithelial sodium channel with 10 µM amiloride, cells pre-treated with DMSO showed only little CFTR function in response to the membrane permeant cAMP analog (CPT-cAMP) at 100 µM and subsequent addition of 1 µM potentiator VX-770 to maximally activate F508del-CFTR [36]. This is pointed out by the small current increase in Fig. 3a, due to the limited number of channels present in the membrane of these cells which were not treated with VX-809, and is also supported by the tiny drop in transepithelial current upon CFTR inhibition, due to administration of the selective channel blocker CFTR_Inh_-172 at 10 µM concentration. Differently, when cells were corrected with VX-809, a substantial rise of current intensity was recorded after addition of CPT-cAMP and each Esc peptide (Fig. 3c and d) which is comparable to the current increase elicited by administration of CPT-cAMP and VX-770 (Fig. 3b), highlighting a consistent CFTR rescue activity. For each sample group, the difference between the current intensity measured before and after CFTR inhibition (ΔI) was calculated (Fig. 3e). Note that in these experiments with primary airway epithelial cells that represent a more suitable in vitro lung model to reflect in vivo situation, the clinically used potentiator VX-770 was used (instead of GEN) and found to display a similar effect to that of the two Esc peptides (Fig. 3e).

In comparison, when epithelia were incubated with each Esc peptide for 24 h in the absence of VX-809, and subsequently exposed to CPT-cAMP and VX-770, a negligible recovery of CFTR function was detected (Fig. S1), suggesting that both peptides lack of corrector activity.

### The potentiator effect of Esc peptides is highly dependent on their primary structure

**Fig. 4:**
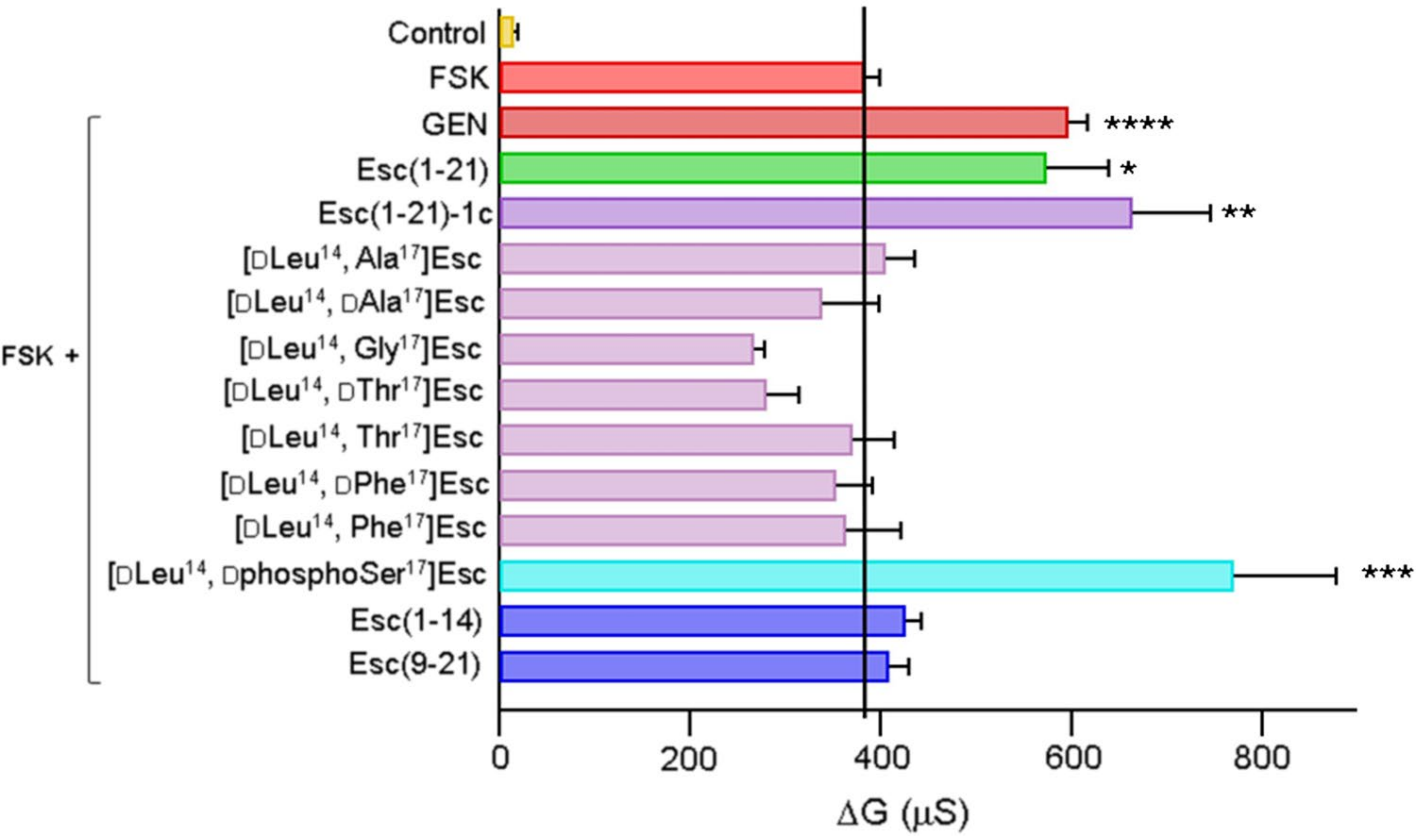
Effect of different analogs of Esc peptides on the CFTR-dependent transepithelial conductance in corrector-rescued F508del-FRT cells. All Esc peptides and GEN were assayed at 10 µM in the presence of 20 µM FSK. Cells pre-incubated with VX-809 but not activated were used as control. Samples pre-incubated with VX-809 and then treated with FSK alone were included for comparison. Data are expressed as mean ± SEM, from n ≥ 6 independent experiments. The level of statistical significance of samples *versus* FSK is indicated as follows: *p < 0.05; **p < 0.01; ***p < 0.001; ****p < 0.0001. Comparison between data was done using Student’s *t* test. The solid black line highlights the delta conductance measured in epithelia treated with 20 µM FSK alone

To explore whether the potentiator effect of Esc peptides relied on their primary structure, and particularly on the presence of Ser^17^ and its putative phosphorylation by intracellular kinases, a series of peptide analogs bearing different d/l amino acids at position 17, were synthesized (Table 1), and used for TEER experiments in VX-809-corrector rescued F508del-FRT cells: peptides carrying l or d-alanine; a peptide with the achiral glycine; peptides bearing another potential target amino acid for protein kinases, i.e., d or l-threonine; and peptides where Ser^17^ was replaced by a residue with a bulky side chain, i.e., d or l phenylalanine. An analog with d-phosphoSer at position 17 was included in the test set for comparison. Figure 4 shows the difference of F508del-FRT transepithelial conductance measured after 10-min stimulation of CFTR with FSK alone or with FSK plus 10 µM of each peptide, and the conductance measured after inhibition of CFTR (upon addition of PPQ102). Except for peptide [dLeu^14^, dphosphoSer^17^]Esc, treatment with all the other isoforms did not significantly raise the function of F508del-CFTR. Interestingly, the effect provoked by [dLeu^14^, dphosphoSer^17^]Esc was stronger than that of the two Esc peptides or GEN and about twofold higher than that elicited by FSK. Subsequently, we also investigated whether shorter portions of Esc peptides were sufficient to preserve CFTR potentiation by performing TEER experiments with the 1–14 and 9–21 fragments of Esc(1–21). No variation in the CFTR-mediated conductance was observed (Fig. 4), meaning that a full-length Esc peptide is necessary to regain the function of F508del-CFTR.

**Table 1:**
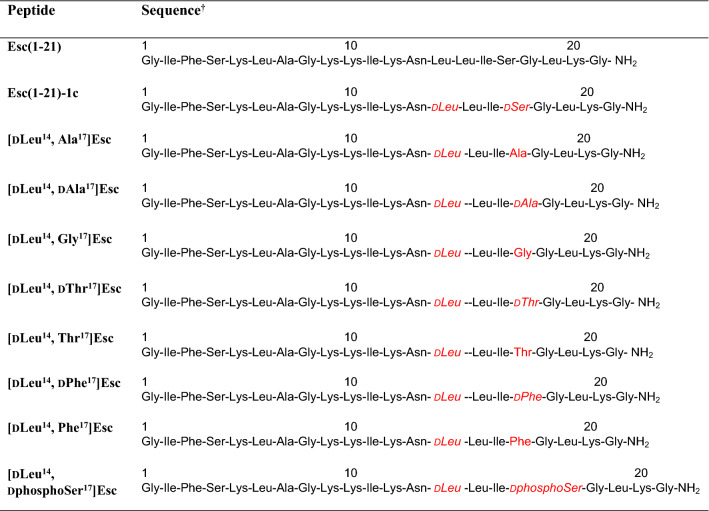
Primary structure of analogs of Esc peptides

^†^Amino acids substitution compared to Esc(1–21) are indicated in red. Amino acids in d configuration are in italics

### Esc peptides modulate F508del-CFTR-mediated chloride current

**Fig. 5:**
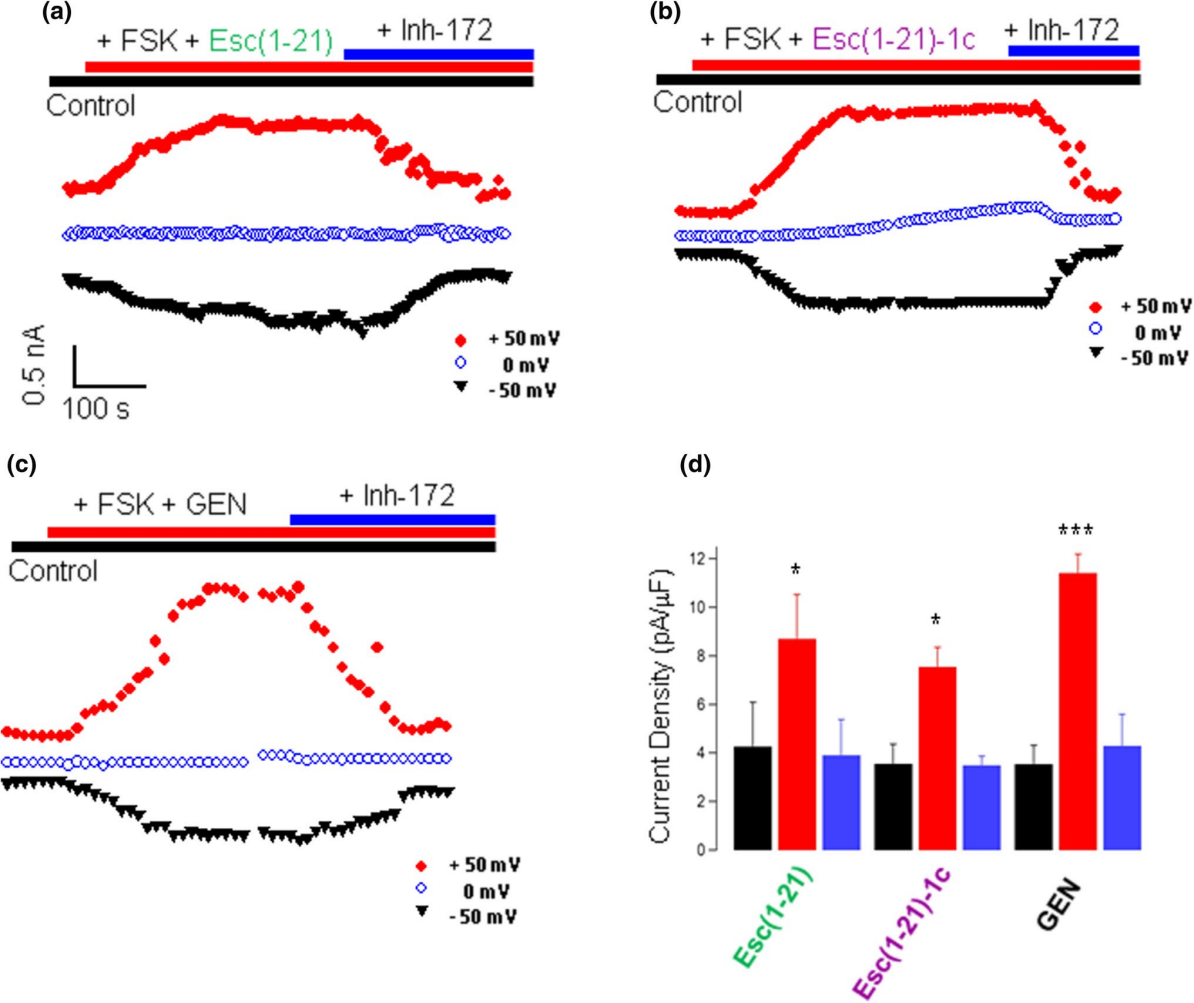
Whole-cell membrane time course in corrector-rescued F508del-FRT cells. **a**–**c** Time course of currents elicited in F508del-FRT cells during the addition of 20 µM FSK + 10 µM Esc peptides (**a** and **b**) or GEN (**c**) and after subsequent application of 10 µM CFTR_Inh_-172, at different potentials, as indicated. **d** Current density measured at + 100 mV for indicated treatment (control basal condition, black bars; after CFTR activation, red bars and after CFTR inhibition, blue bars). Data are expressed as mean ± SEM from n ≥ 3 independent experiments. The level of statistical significance of samples *versus* the control basal condition is indicated as follows: *p < 0.05; ***p < 0.001. Comparison between data was done using Student’s *t* test

To get insight into the mechanism of CFTR activation by Esc peptides, patch-clamp experiments were carried out on VX-809-rescued F508del-FRT cells. In whole-cell recordings, CFTR was activated by FSK in combination with Esc peptides or GEN that were added to the extracellular solution. Representative images of superimposed currents elicited at membrane potentials in the range from  – 80 mV to + 120 mV in the control external solution, as well as after addition of FSK plus Esc peptide/GEN and subsequent administration of CFTR_Inh_-172 are reported in Fig. S2. The time-course of membrane currents during experiments is plotted in Fig. 5 (a–c). The presence of the peptides at the extracellular side of the membrane patch induced ~ twofold increase of the current amplitude at + 100 mV compared to the control basal condition (Fig. 5d), pointing out a substantial recovery of the mutated channel activity. This effect was comparable to that provoked by GEN (Fig. 5c and d). Importantly, when the CFTR_Inh_-172 was added, the current amplitude reverted to the value measured before CFTR activation, confirming that the current increase induced by the peptides in combination with FSK is due to activation of CFTR (Fig. 5 and Fig. S2).

As further investigation, the same experiments were performed with untransfected FRT cells which do not express endogenous CFTR (null-FRT). There was no significant change of ion current upon addition of FSK plus GEN or FSK plus Esc peptides (Fig. S3), highlighting a specific action of peptides on CFTR channel.

**Fig. 6:**
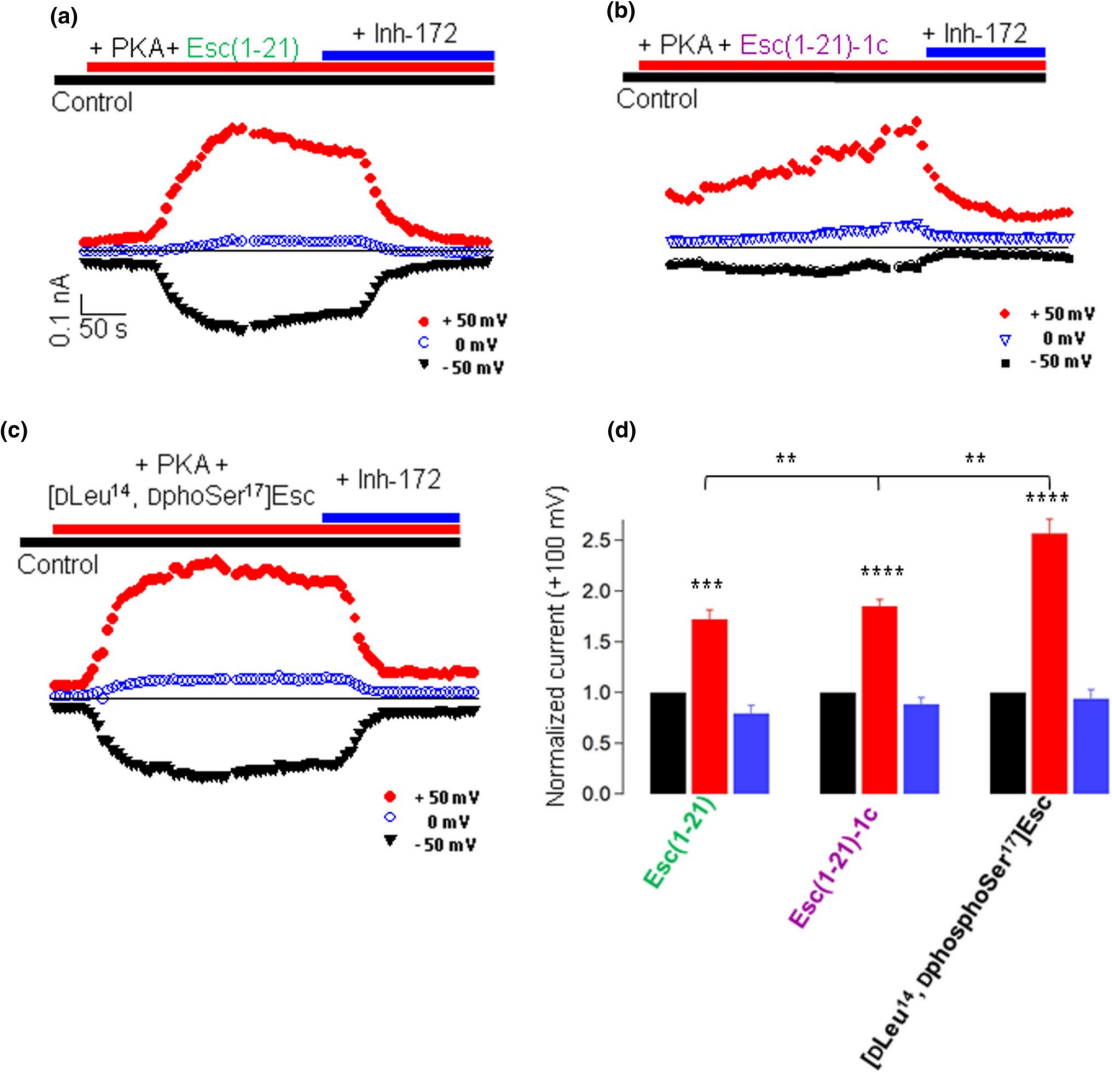
Analysis of membrane currents in F508del-FRT cells from inside-out patch-clamp. **a**–**c** Time course of current elicited in F508del-FRT cells by the application of a bath solution containing PKA + 10 µM peptide and after the addition of CFTR_Inh_-172. Currents were activated by pulses 50 ms long, administrated every 5 s at the indicated voltages. **d** Current ratio at + 100 mV for indicated treatment (control basal condition, black bars; after CFTR activation by peptides + PKA, red bar; and after CFTR inhibition, blue bar). Current intensity was normalized to that measured under control basal conditions (before addition of compounds). Data are expressed as mean ± SEM from *n* ≥ 3 independent experiments. The level of statistical significance of samples *versus* the control basal condition, as well as the statistical significance among samples after CFTR activation are indicated as follows: **p < 0.01; ***p < 0.001; ****p < 0.0001. Comparison between data was done using Student’s *t* test

CFTR activation by Esc peptides was also studied under cell-free conditions by the inside-out patch-clamp configuration using large pipette tips to obtain macro-patches containing multiple CFTR channels (Fig. 6). The [dLeu^14^, dphosphoSer^17^]Esc peptide was also included for comparison. As shown by the time course of currents elicited in F508del-FRT cells (current traces are indicated in Fig. S4), after inducing phosphorylation of CFTR by the addition of 1 mM ATP and 125 nM of the catalytic subunit of PKA, the administration of 10 µM peptides in the intracellular bath solution clearly augmented CFTR-mediated current (Fig. 6a–c) with ~ 2.5-fold higher current intensity at + 100 mV for the more active [dLeu^14^, dphosphoSer^17^]Esc peptide, compared to control basal conditions (Fig. 6d). Also in this case, currents activated by the peptides were rapidly blocked by CFTR_Inh_-172 (Fig. 6a–d), supporting the conclusion that activation of CFTR implies direct binding of the peptides to the ion channel. Note that either FSK or PKA-mediated phosphorylation of the mutated channel in the whole-cell and inside-out patch-clamp configurations respectively was not sufficient to significantly activate the channel without the addition of any peptide or potentiators as GEN (Fig. S5).

### Esc peptides potentially interact with F508del-CFTR

The possible interaction between the NBDs of the F508del-CFTR and the peptides Esc(1–21) and [dLeu^14^, dphosphoSer^17^]Esc was investigated by molecular dynamics (MD) simulations. Given the lack of structural information on the possible binding mode of AMPs to the NBDs by structural biology studies, a blind approach was followed. Specifically, the recognition and binding between F508del NBDs and each peptide were simulated without any conformational or positional bias by placing each peptide and the NBDs heterodimer in a random reciprocal orientation within the simulation box, and at a distance higher than 40 Å to each other. Moreover, to enhance the statistical significance of these MD simulations, three independent replicas of 500 ns were simulated for each system, for a total simulation time of 1.5 µs on every peptide [40]. Results unequivocally showed that Esc(1–21) and [dLeu^14^, dphosphoSer^17^]Esc recognize different binding sites on the NBDs, most likely because of their different chemical composition, physicochemical properties, and phosphorylation state.

**Fig. 7:**
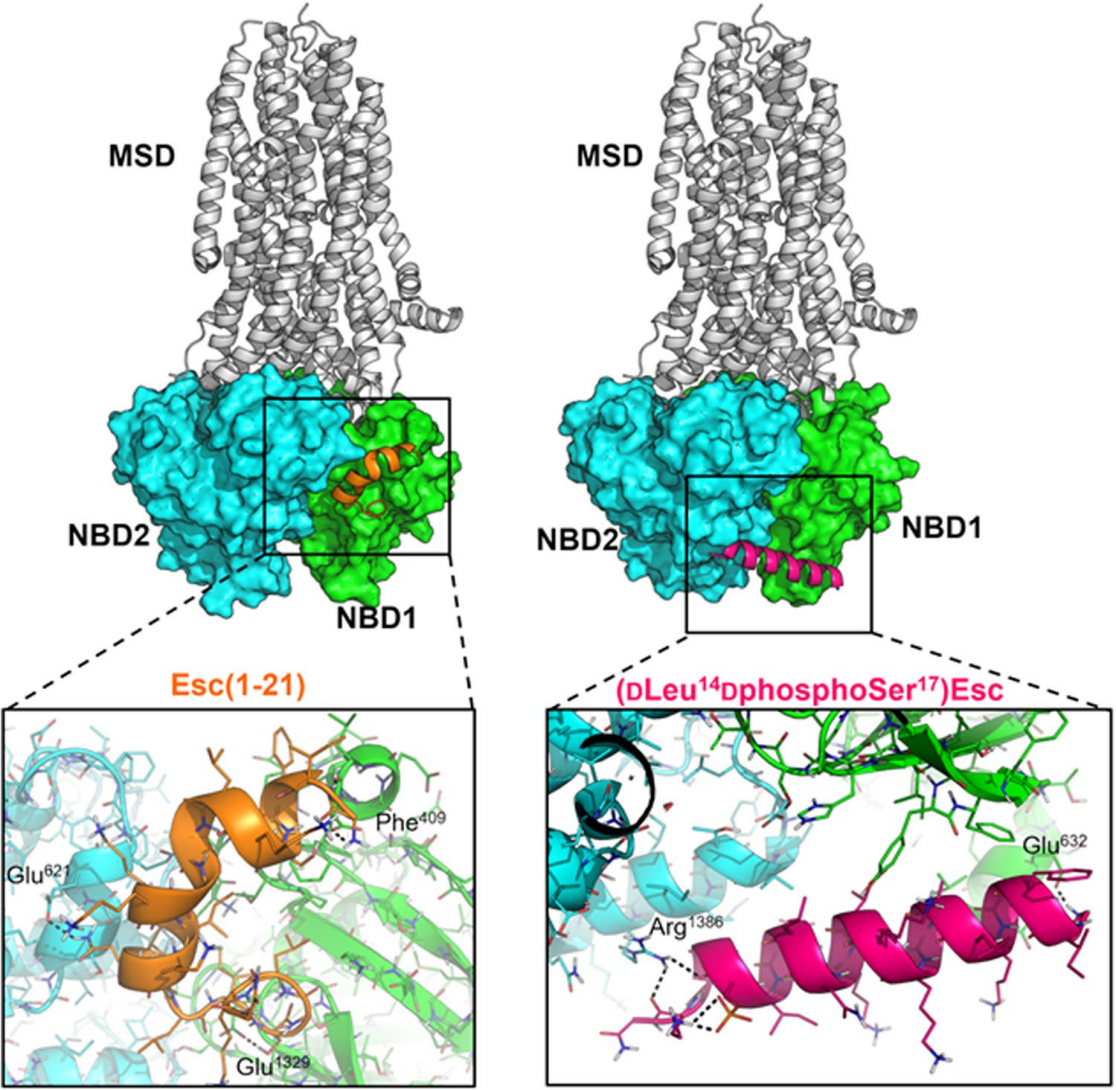
Structural insights into intermolecular recognition and binding of peptides to F508del NBDs by MD simulations. A representative structure of NBDs/peptide interaction as extrapolated from MD trajectories is shown in the top panels. Bottom panels show a magnification of the peptide binding sites with the main H-bonds being highlighted by black dashed lines. Left: Esc(1–21)/NBDs; right: [dLeu^14^, dphosphoSer^17^]Esc/NBDs. The full-length structure of CFTR in complex with ivacaftor coded by PDB-ID: 6O2P [2] is shown in white cartoons for comparison purposes, and to highlight the position of the MSDs in relation to NBDs. The NBD1 is shown as a green surface in the top panels, and as green cartoons and lines in the bottom panels; the NBD2 is shown as a cyan surface in the top panels, and as cyan cartoons and lines in the bottom panels. Esc(1–21) and [dLeu^14^, dphosphoSer^17^]Esc are colored orange and hot pink, respectively

In detail, in every MD replica Esc(1–21) coherently recognizes a binding site located on the lateral edge of NBD1 near its N-terminal tail, in close proximity of the binding interface with the NBD2 (Fig. 7). In contrast, the [dLeu^14^, dphosphoSer^17^]Esc binds near the cytoplasmic side of the NBDs bridging NBD1 with NBD2 in proximity of the cytoplasmic entrance to the pore (Fig. 7). Analysis of the electrostatic surface potential of NBDs on the representative frame extracted from MD trajectories shows that Esc(1–21) exploits a cluster of basic residues located at its N-terminal end to recognize a negatively charged region of NBD1. Differently, the [dLeu^14^, dphosphoSer^17^]Esc peptide recognizes and binds a more hydrophobic region of the NBDs bridging NBD1 with NBD2 (Fig. S6). An in depth visual inspection of the peptides binding mode revealed that the lack of phosphorylation on Ser^17^ in Esc(1–21) enhances the basicity of the Esc(1–21) peptide, which in turn binds an acidic region of the NBDs where a relevant charge complementarity with the peptide can be observed (Fig. S6). In [dLeu^14^, dphosphoSer^17^]Esc, the phosphorylated Ser^17^ performs a highly persistent intramolecular salt-bridge (average persistence in MD replicas is around 88%) with Lys^20^, which formally decreases the overall basicity of the peptide and sequestrates the basic residue from its potential interaction with the NBDs. Accordingly, MD simulations suggested that [dLeu^14^, dphosphoSer^17^]Esc preferentially binds into a more hydrophobic binding site of the NBDs compared to Esc(1–21). Besides, Esc(1–21) interacts mostly with the NBD1 by establishing a network of H-bond interactions with the backbone of Phe^409^, Glu^621^, and Glu^1329^, as well as hydrophobic interactions with Phe^405^, Phe^433^, Thr^438^, and Pro^439^ through the lipophilic edge of the peptide formed by Phe^3^, Ile^11^, Leu^14^, Ile^16^ and Leu^19^ (Fig. 7). The [dLeu^14^, dphosphoSer^17^]Esc binds with a similar polarity but, compared to Esc(1–21), it is equally distributed within the surface of NBD1 and NBD2. The peptide establishes H-bond interactions with Glu^632^ and Arg^1386^, while the non-polar edge of the peptide establishes an extended network of hydrophobic interactions with Ile^618^, Tyr^625^, Phe^626^, Leu^636^, Pro^1378^, Val^1379^, and Ile^1383^ from the NBD1/NBD2 interface (Fig. 7).

The theoretical affinity of the two AMPs investigated by MD simulations was estimated by the Molecular Mechanics Generalized Born Surface Area (MM-GBSA) approach along MD trajectories [41]. Results unequivocally show that the [dLeu^14^, dphosphoSer^17^]Esc binds the NBDs with a stronger theoretical affinity than the Esc(1–21) (ΔE_binding_ of Esc(1–21) = -22.65 ± 0.58 kcal/mol; ΔE_binding_ of [dLeu^14^, dphosphoSer^17^]Esc = -29.07 ± 0.69 kcal/mol), which suggests that phosphorylation of Ser^17^ coupled with the introduction of dLeu at position 14 might enhance the peptide’s affinity for the F508del NBDs. Coupling experimental evidence with computational details also led to hypothesize that the different binding sites recognized by Esc(1–21) and [dLeu^14^, dphosphoSer^17^]Esc and the corresponding different theoretical affinity might play a role in gating the chloride channel pore. These structural insights prove useful to rationalize experimental evidences described in this work, as well as in the design and prioritization of further series of AMPs and peptide-mimetics in a drug development perspective.

## Discussion

The principal issue in CF patients is the reduced conductance of chloride and bicarbonate ions across defective CFTR in the apical membrane of epithelial cells, with abnormal lung secretions and loss of respiratory function, which is still fatal in early adulthood [42, 43]. Remarkably, even in the era of CFTR modulation therapies, management of lung infections remains challenging, especially for patients with advanced stages of lung disease [44, 45]. In this work, we discovered that in contrast to the clinically used CFTR modulators, Esc peptides would give particular benefit especially to CF sufferers with *P. aeruginosa* lung infection, by combining their antibacterial activity with a potentiating effect of CFTR. The finding of (i) a rapid raise of ion conductance when each Esc peptide was added to corrector-rescued F508del-CFTR expressing epithelium in the presence of FSK and (ii) the return of such conductance to the initial value (measured before CFTR activation), after administration of the selective CFTR inhibitor, clearly proved that the conductance increase stimulated by Esc peptides is mediated by the activated CFTR. Such findings suggested the capability of Esc peptides to augment the ion permeability of the rescued-F508del-CFTR by presumably modifying the channel open-closed probability, like for GEN. This was then confirmed by patch-clamp experiments showing the peptides’ ability to increase the ion current through F508del-CFTR. Furthermore, electrophysiological experiments and computational studies have underscored how the potentiator effect of Esc(1–21) and its diastereomer is (i) a highly specific process relying on their primary structure and particularly on the presence of Ser^17^; and (ii) the result of a direct interaction of the peptides with CFTR, as proposed for other CFTR activators, including GEN [38, 46, 47]. Computational studies suggested that a direct interaction between peptides and NBDs of F508del CFTR does occur, although in different sites depending on the phosphorylation state of the peptide at Ser^17^. Esc(1–21) binds preferentially within a negatively charged region at the N-terminal of NBD1, whereas the diasteromeric form phosphorylated at Ser^17^ (i.e., [dLeu^14^, dphosphoSer^17^]Esc) binds preferentially near the cytoplasmic side of the ionic pore bridging NBD1 and NBD2, thus possibly promoting the stabilization of their heterodimeric form. Moreover, the Esc(1–21) peptide is endowed with a weaker theoretical affinity for the NBDs compared to [dLeu^14^, dphosphoSer^17^]Esc.

Our hypothesis is that Esc peptides can interact directly with CFTR from the cytosolic site, upon entrance into epithelial cells. Once in the cytoplasm, the diastereomer Esc(1–21)-1c in particular could be phosphorylated at the level of d-Ser, thus switching to the more efficient form to improve CFTR pore opening. However, we cannot exclude the potential interaction of Esc peptides with the extracellular domain of CFTR, while in turn we can rule out that the peptides work as CFTR correctors. Indeed, as shown by Ussing experiments, there was no increase in CFTR-mediated current upon addition of CPT-cAMP and VX-770 to the epithelium from a homozygous F508del patient preincubated with each Esc isomer rather than with VX-809.

Furthermore, we demonstrated that the two Esc peptides do not alter the airway epithelial barrier permeability or the paracellular ions transport. This was indicated by the high level of transepithelial electrical resistance (low conductance) upon 24-h treatment of CFBE41o- and FRT epithelia with Esc(1–21) or its diastereomer Esc(1–21)-1c, meaning that cell junctions remain well tightened.

Many proof-of-concept studies have disclosed that chemically modified ATP analogs and small organic molecules (e.g., isoflavones) have good potentiation effect by enhancing CFTR channel gating. However, the expected unspecific binding of ATP analogs to other proteins implicated in physiological functions limits the development of these molecules to the clinic. Other compounds capable of improving the activity of mutant CFTR can promote its phosphorylation by inhibiting phosphodiesterase’s activity, to heighten the intracellular level of cAMP. Ivacaftor was the first CFTR potentiator approved for clinical application [48]. It works upon binding at the interface of the two CFTR’s MSDs [49]; however, ivacaftor-based treatment results in minor benefit to patients [48] with only 10% enhanced lung function [50]. Other potentiators have been identified and are now under preclinical characterization or in drug development pipeline [51–53], but some of them give off-targets effects [54]. Despite the current ivacaftor/lumacaftor combination therapy (Orkambi and more recently Trikafta from Vertex Pharmaceuticals) designed for CF patients with the genetic F508del mutation in homozygosity have a good efficacy [20], treatment of lung pathology in CF would take advantage from antimicrobial compounds with a repairing action of the lung epithelium in addition to a potentiation of CFTR activity. All these activities are covered by Esc peptides and are of remarkable relevance, due to the occurrence of chronic infections in CF lungs and to the impaired re-epithelialization of airway wounds in CF patients [55]. Overall, our findings concur to emphasize Esc peptides as attractive novel agents for treatment of CF lung pathology, likely as adjuvants of clinically used CFTR modulators, upon inhalation.

In recent years, the employment of peptides as candidates for clinical translation has gained a raising interest, mainly due to their high specificity, safety, and tolerability [21, 56, 57]. Nowadays, there are more than 50 commercially available peptide-based medicines [58], while hundreds of them are under preclinical and clinical experimentation for the development of new drugs, including those aimed at treating respiratory infections, either following intravenous injection (i.e., POL7080) or inhalation. This latter is the most advantageous administration route to maximize drug efficacy for treatment of lung infections and to reduce side effects of drugs, compared to oral and/or i.v. injection [57, 59]. Peptide-based drugs marketed as inhaled medications for CF, upon nebulization, encompass the recombinant version of the human DNaseI (Pulmozyme®) to decrease mucus viscosity [60]. Unfortunately, peptides themselves can be difficult to handle because of their susceptibility to proteases of body fluids and because of their interaction with biological surfaces that might prevent them from reaching the target site. Nevertheless, we newly showed how polyvinyl alcohol engineered poly(lactic-co-glycolic) acid (PLGA) nanoparticles represent an enticing nano-formulation for delivery of Esc peptides in the conductive airways, extending their efficacy against Pseudomonas-induced pneumonia compared to the free peptides [61]. A gradual release of nano-entrapped AMPs should restrict the frequency of peptide administration, while ameliorating patients’ quality of life and compliance to prescribed treatments. Studies performed with the tetra branched AMP M33 have shown that its packing into nano-systems lowers its toxicity [62], while encapsulation of M33 into nanoparticles lengthens lung residence time of the peptide administered via aerosol [63].

Considering our promising experimental data, a valuable therapeutic strategy to counter lung pathology in CF could lean on the development of proper inhaled biodegradable PLGA nanoparticles loaded with Esc peptides, over nebulization or dry-powder formulations. Globally, this work has provided the first evidence that AMPs, in the case of Esc peptides, can affect the activity of F508del-CFTR upon direct interaction with both NBD1 and NBD2 near the cytoplasmic side of the ionic pore. This feature has not been explored previously with any AMP and highly contributes to stress the usefulness of these peptides as ideal pharmaceuticals for treatment of CF pulmonary disease. Remarkably, they would have the capability to act not only as antibiotics against lung infections limiting the induction of bacterial resistance, and as promoters of airway wound repair, which is delayed in CF patients [64, 65], but also as CFTR modulators to ameliorate the conductance of the mutated CFTR channel.

In summary, we report herein a novel property of AMPs, and peptides in general, that is the potentiator activity of CFTR. The existence of multiple features of AMPs will likely offer the possibility of a new up-and-coming pharmacological approach to address the functional defect in CF sufferers and its associated harmful effects.

## Materials and methods

### Cells and peptides

For our experiments, we used Fischer rat thyroid (FRT) and CFBE41o- cells without or with stable expression of mutated or wild-type CFTR (F508del-FRT and wt-FRT; F508del-CBFE41o- and wt-CFBE41o-, respectively) [66]. FRT cells were cultured in Coon’s modified Ham’s F-12 medium (Sigma-Aldrich, St. Luis, MO) and CFBE41o- cells were cultured in minimal essential medium (MEM) both supplemented with 10% fetal calf serum, 2 mM L-glutamine, 100 U/ml penicillin, and 100 μg/ml streptomycin. Primary human bronchial epithelial cells derived from CF and non-CF individuals were provided by the Italian Cystic Fibrosis Foundation (FFC) Cell Culture Service. They were cultured in flasks in proliferative serum-free medium containing 1:1 mixture of RPMI 1640 and LHC basal medium (Life Technologies, Monza, Italy) supplemented with various hormones and supplements, 100 U/ml penicillin and 100 μg/ml streptomycin. Synthetic Esc peptides and analogous were purchased from Biomatik (Wilmington, USA).

### TEER to evaluate lung epithelial integrity and CFTR activation

To evaluate the lung epithelia integrity, FRT and CFBE41o- cells expressing mutated or functional CFTR were seeded on permeable supports (24 Millicell plates PSHT01QR1) and incubated for 24 h in their standard culture medium in the presence of peptides, at different concentration. DMSO, 0.1% v:v, final concentration) that was used to solubilize each compound was included as control [31]. After 24 h, the medium was replaced with saline solution containing (in mM): 130 NaCl, 2.7 KCl, 1.5 KH_2_PO_4_, 1 CaCl_2_, 0.5 MgCl_2_, 10 glucose, 10 Na-Hepes (pH, 7.4). Saline solution was added to both apical and basolateral compartments of the permeable supports. Samples were incubated for 10 min at 37 °C and TEER was measured in basal conditions without CFTR activation, with the epithelial voltmeter (MILLICELL ERS-2, Millipore Burlington, MA).

To evaluate CFTR activation, the same saline solution was used. Cells expressing F508del mutation were incubated with 1 µM VX-809 to favor the mutant protein rescue. After measurement of basal TEER, 20 µM FSK + 10 µM GEN as positive control, or FSK + peptides at the desired concentration were added on the epithelium apical side. Finally, 30 µM PPQ102 was added on the epithelium apical side to block CFTR, so to measure the own activity of CFTR. TEER measurement was performed at 37 °C after 10 min from the addition of each compound. From the values of TEER measured before and after CFTR inhibition, we calculated the CFTR-dependent TEER for each condition. All values of TEER were converted to transepithelial conductance (TEEC) using the formula TEEC = 1/TEER [67].

### Ussing chamber (Short-Circuit Current Recordings)

Primary human bronchial epithelial were seeded on porous membranes (12 mm Snapwell inserts, Corning, code 3801) as previously described [68] to form a differentiated epithelium under air–liquid condition. Epithelia were treated for 24 h with 1 µM VX-809 or 0.1% DMSO. Afterwards, epithelia were mounted in a vertical diffusion chamber, resembling a Ussing chamber with internal fluid circulation. Both apical and basolateral hemi chambers were filled with 5 ml of a solution containing (in mM): 126 NaCl, 0.38 KH_2_PO_4_, 2.13 K_2_HPO_4_, 1 MgSO_4_, 1 CaCl_2_, 24 NaHCO_3_, and 10 glucose. Both sides were continuously bubbled with a gas mixture containing 5% CO_2_—95% air and the temperature of the solution was kept at 37 °C. The transepithelial voltage was short-circuited with a voltage-clamp (DVC-1000, World Precision Instruments) connected to the apical and basolateral chambers via Ag/AgCl electrodes and agar bridges (1 M KCl in 1% agar).

The short-circuit current was recorded with a PowerLab 4/25 (AD Instruments) analog-to-digital converter connected to a computer. Afterwards, 10 µM amiloride was added; subsequently, CFTR channels were activated by phosphorylation upon addition of 100 µM CPT-cAMP (permeable cAMP analog). Then, 1 mM of the potentiator VX-770 as well as each Esc peptide (10 µM) was added on the epithelium apical side. Finally, 10 µM of CFTR_Inh_-172 was added to the epithelium apical side to measure the own activity of CFTR. Current measurements were done after 5 min from the addition of each substance [69].

### Patch-clamp experiments

Whole-cell and inside-out membrane currents were recorded in FRT cells stably expressing F508del CFTR after incubation with 1 µM of the corrector VX-809 for 24 h to increase mutated CFTR membrane expression.

For whole-cell experiments the extracellular (bath) solution contained (in mM): 150 NaCl, 1 CaCl_2_, 1 MgCl_2_, 10 glucose, 10 mannitol, 10 Na-HEPES (pH 7.4). The pipette (intracellular) solution contained (in mM): 120 CsCl, 10 TEA-Cl, 0.5 EGTA, 1 MgCl_2_, 10 Cs-HEPES, 40 mannitol, 1 ATP. To activate CFTR, 20 µM FSK was added to the extracellular solution to phosphorylate the channel. In some samples the combination FSK + 50 µM GEN (positive control) as well as FSK + 10 µM peptides were added to the extracellular solution to test their potentiator activity.

For inside-out patch-clamp experiments, the pipette solution contained (in mM): 150 N-methyl-D-glucamine chloride (NMDG-Cl), 3 CaCl_2_, 2 MgCl_2_, 10 Na-Hepes (pH 7.3). The bath solution for these experiments contained (in mM): 150 NMDG-Cl, 2 MgCl_2_, 10 EGTA, 10 Na-Hepes, 1 ATP, which was supplemented with 125 nM catalytic subunit of PKA (Promega, Sunnyvale, CA) and peptides at 10 µM. Pipette electrical resistance for both whole-cell and inside-out experiments was 3–5 MΩ.

For both patch configurations, the protocol to correlate current intensity and voltage for stimulation consisted of 500-ms voltage steps from -100 to + 100 mV or from -80 to + 120 mV in 20 mV increments, starting from a holding potential of -60 mV. The time-dependence of CFTR activation and inactivation was monitored by application of steps of 50 ms pulses to − 100, − 50, 0, 50 and 100 mV every 5 s from a holding potential of − 60 mV. The interval between steps was 4 s. Membrane currents were filtered at 1 kHz and digitized at 5 kHz by a low–pass 4–pole Bessel filter.

### Computational studies

The initial rough structure of the F508del form of NBDs was generated by homology modelling with Prime (Schrodinger, LLC, New York) [70] using as a template the 3.2 Å resolution electron microscopy structure of the human phosphorylated ATP-bound CFTR (PDB-ID: 6MSM) [71]. To decrease the complexity of the macromolecular target, the MSD were removed. The resulting F508del NBDs structure was included in a rectangular box of explicit TIP3P type water molecules buffering 10 Å from the protein surface. Histidine protonation states were assigned by the H +  + server, 3.2 version [72],while the total charge of the system was neutralized by the addition of 8 Cl^−^ counter-ions. The system was then relaxed through energy minimization and MD simulations with Amber18 [73, 74] using the following procedure: (i) water molecules and ions were first relaxed by 500 steps with the steepest descent algorithm (SD) followed by 2500 steps with the conjugate gradient algorithm (CG); (ii) the solvated solute was relaxed by 1000 steps with the SD and 5000 steps with the CG, before (iii) being heated from 0 to 300 K during 1 ns of MD using the Langevin thermostat at constant volume; (iv) system’s density was equilibrated by the Berendsen barostat for 1 ns at constant pressure; (v) a first unrestrained MD of 50 ns was run at constant pressure, before (vi) the final production of MD lasting 500 ns. The ff14SB force field was used to parametrize the protein, while the General Amber Force Field (GAFF) was used for ATP molecules [75]. The representative structure of the F508del NBDs was extrapolated from MD trajectories by cluster analysis with the CPPTRAJ software [76].

A Nuclear Magnetic Resonance (NMR) reference structure of Esc peptides was retrieved from the Protein Databank (www.rcsb.org/pdb) under the PDB-ID 5XDJ [77]. Parameters for d-amino acids were adapted from those implemented in Amber18 for l-amino acids, while parameters for the phosphoserine were retrieved from the AMBER parameter database (Bryce group) [78], as they have been recently tested and validated [79].

The recognition and binding of peptides was simulated using the MD procedure described above, by placing each peptide and the F508del NBDs in the simulation box at a distance higher than 40 Å and in a random reciprocal orientation. Three independent MD replicas of 500 ns were run for each system. The electrostatic surface potential was calculated by the Adaptive Poisson-Boltzmann Solver (APBS) [80].

### Statistical analysis

Quantitative data derived from independent experiments were expressed as the mean ± standard error of the mean (SEM). Statistical significance was determined using Student’s t test, with Igor environment (Wavemetrics, Lake Oswego, OR). Comparison between data was done using Student’s t test, after controlling the normal distribution of data with the Anova normality test. *P* values of < 0.05 were assumed to be statistically significant. The levels of statistical significance are indicated in the legend to figures.

## Supplementary Information

Below is the link to the electronic supplementary material.

Supplementary file1 (PDF 622 KB)

## Data Availability

All data are available in the manuscript and in the Supplementary Information.
