# Supplementary material for: Esc peptides as novel potentiators of defective cystic fibrosis transmembrane conductance regulator: an unprecedented property of antimicrobial peptides
Source: Cell Mol Life Sci. 2021 Dec 31;79(1):67. doi: 10.1007/s00018-021-04030-2 (PMC8752549; doi:10.1007/s00018-021-04030-2)
Supplement: Supplementary file 1 — Supplementary file1 (PDF 622 KB) [file 18_2021_4030_MOESM1_ESM.pdf]

Supplementary Information

Esc peptides as novel potentiators of defective cystic fibrosis transmembrane conductance regulator: an unprecedented property of antimicrobial peptides

Loretta Ferrera[1*], Floriana Cappiello[2], Maria Rosa Loffredo[2], Elena Puglisi[2], Bruno Casciaro[3], Bruno Botta[4], Luis J.V. Galietta[5,6], Mattia Mori[7*], Maria Luisa Mangoni[2*]

[1]U.O.C. Genetica Medica, Istituto di Ricovero e Cura a Carattere Scientifico (IRCCS) Istituto Giannina Gaslini, 16147 Genoa, Italy

[2]Department of Biochemical Sciences, Laboratory affiliated to Istituto Pasteur Italia-Fondazione Cenci Bolognetti, Sapienza University of Rome, Rome 00185, Italy

[3]Center for Life Nano- & Neuro-Science, Fondazione Istituto Italiano di Tecnologia (IIT), Rome 00161, Italy

[4]Department of Chemistry and Technology of Drugs, "Department of Excellence 2018−2022", Sapienza University, P. le Aldo Moro 5, 00185 Rome, Italy

[5]Telethon Institute of Genetics and Medicine (TIGEM), 80078 Pozzuoli (NA), Italy

[6]Department of Translational Medical Sciences, University of Napoli "Federico II", 80131 Napoli, Italy

[7]Department of Biotechnology, Chemistry and Pharmacy, "Department of Excellence 2018−2022", University of Siena, 53100 Siena, Italy

* Corresponding authors:
Loretta Ferrera; Email: loretta.ferrera@unige.it
Mattia Mori; Email: mattia.mori@unisi.it
Maria Luisa Mangoni; Email:marialuisa.mangoni@uniroma1.it

This PDF file includes:

Supplementary Text
Figs. S1 to S6

Supplementary Text

Null FRT cells

Null FRT cells were cultured in Coon's modified Ham's F-12 medium supplemented with 10% fetal calf serum, 2 mM L-glutamine, 100 U/ml penicillin, and 100 μg/ml streptomycin.

Short-Circuit Current Recordings in primary bronchial epithelium.

Primary human bronchial epithelial were seeded on porous membranes (12 mm Snapwell inserts, Corning, code 3801) as described in the main text to form a differentiated epithelium under air-liquid condition. Epithelia were treated for 24 h with 0.1% DMSO as well as with 1 μM VX-809 or 10 μM Esc peptide. Epithelia were mounted in a vertical diffusion chamber, as described in the main text. The transepithelial voltage was short-circuited with the same voltage-clamp and the short-circuit current recorded as described in the main text. Afterwards, 10 μM amiloride was added; subsequently, CFTR channels were activated by phosphorylation upon addition of 100 μM CPT-cAMP. Then, 1 μM of the potentiator VX-770 was added on the epithelium apical side. Finally, 10 μM $CFTR_{Inh}$-172 was added to the epithelium apical side. Current measurements were done after 5 min from the addition of each substance.

Patch-clamp experiments in null FRT cells

Whole-cell membrane currents were recorded in null FRT cells not expressing CFTR by using the same experimental solutions and stimulation protocols as described in the main text for F508del- FRT.

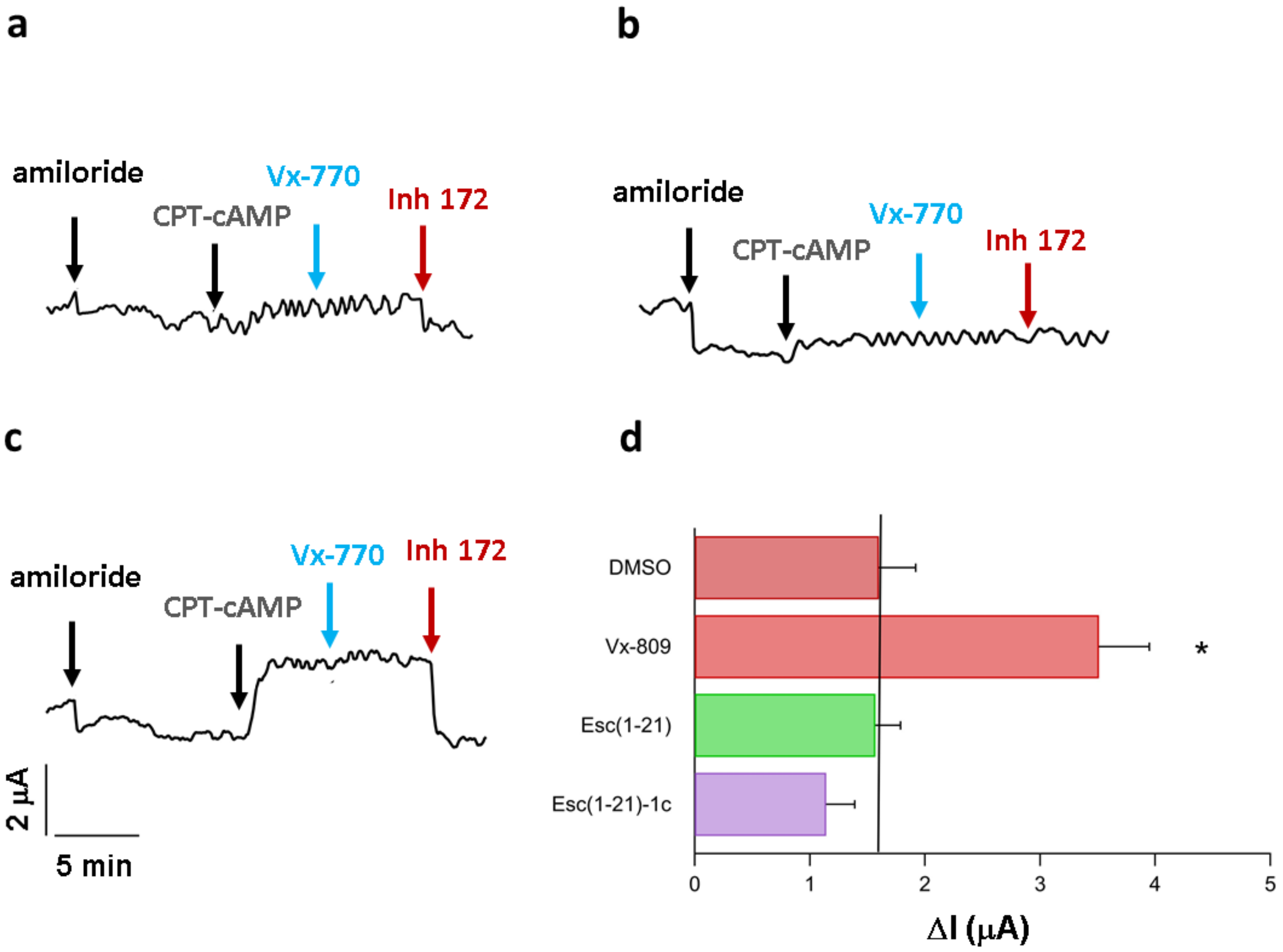

Fig. S1 Effect of peptides on F508del-CFTR activity in primary airway epithelial cells by the short-circuit current technique. **a-c** Representative traces from Ussing chamber recordings of bronchial epithelial cells (HBE) derived from a homozygous F508del patient after 24 h incubation with 0.1% DMSO and subsequent treatment with CPT-cAMP +VX-770 (a) or after incubation with 10 µM Esc(1-21) (b) or 1 µM VX-809 (c) and subsequent treatment with CPT-cAMP +VX-770. Pre-incubation of samples with Esc(1-21)-1c gave similar results to those of Esc(1-21) and therefore they are not shown. **d** Bar graphs indicate the CFTR-mediated current as the difference between the current measured in the epithelia treated with the indicated compounds before and after inhibition of CFTR by $CFTR_{Inh}$-172 (10 µM). Data are the mean ± SEM from n=3 independent experiments. The level of statistical significance *versus* DMSO-treated samples is indicated as follows: *$p<0.05$. Comparison between data was done using Student's t test. The solid black line indicates the delta current measured in epithelia treated with DMSO

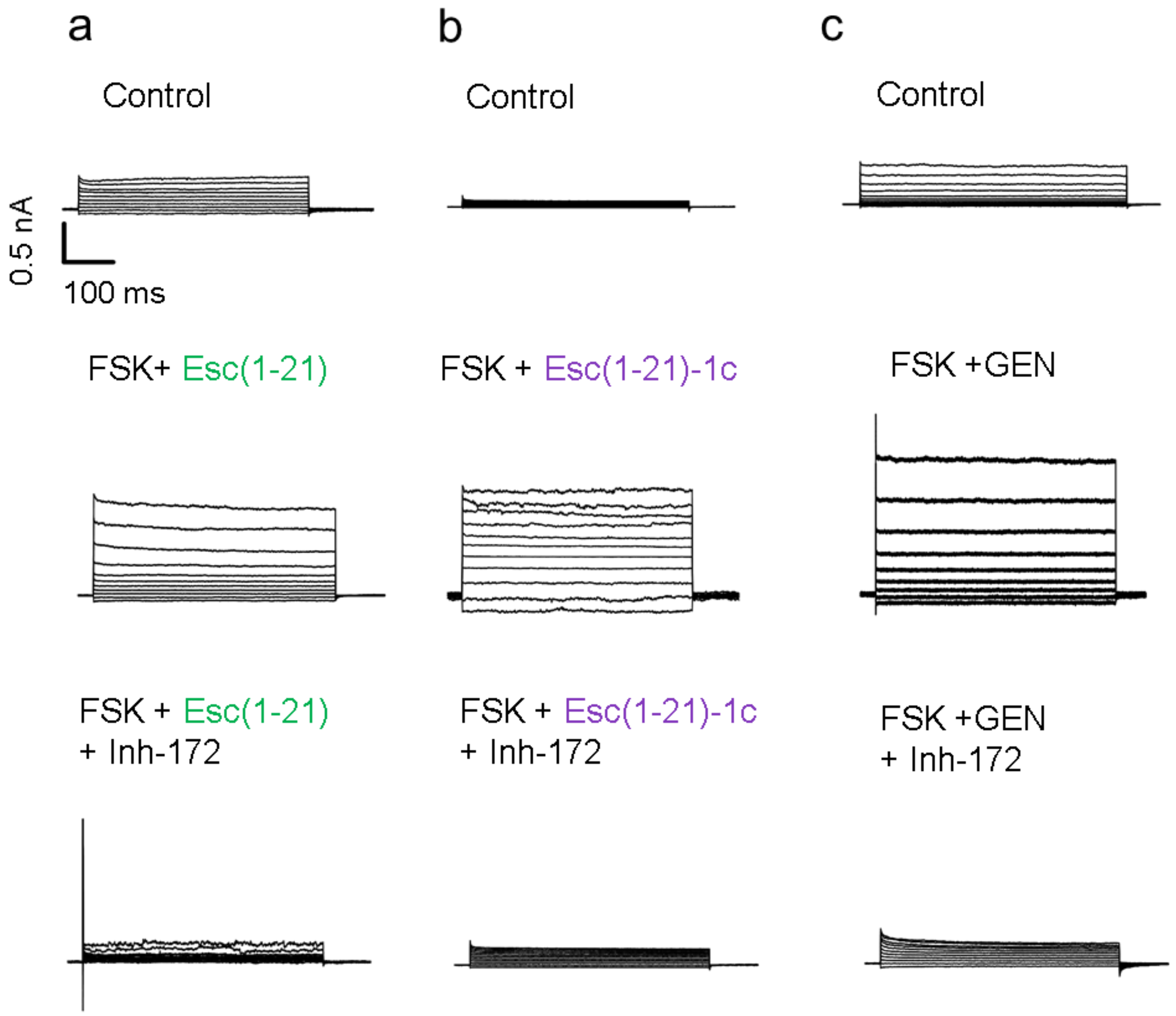

Fig. S2 Analysis of membrane patch in F508del-FRT cells from whole-cell patch technique. **a** Representative superimposed currents elicited at membrane potential from -80 to +120 mV in the control solution (top panel); after addition of 20 μM FSK+10 μM Esc(1-21) (central panel) and after subsequent addition of 10 μM $CFTR_{Inh}$-172 (bottom panel). **b** The same as **a** but for 10 μM Esc(1-21)-1c. **c** The same as **a** but for 50 μM GEN

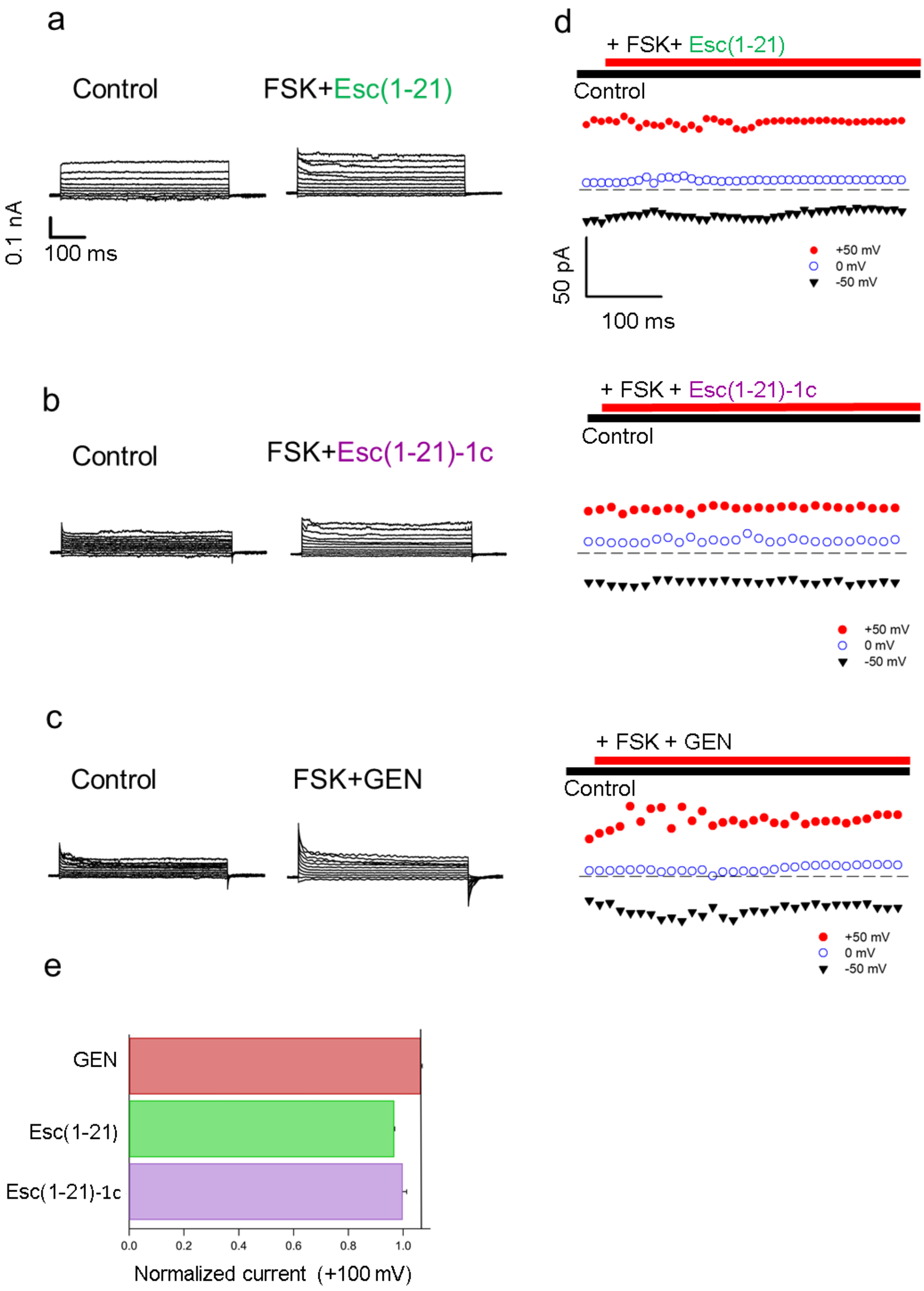

Fig. S3 Membrane currents elicited in null FRT cells. **a-c** Representative whole-cell membrane currents from whole-cell patch-clamp experiments. The figure shows superimposed currents elicited at membrane

potentials in the range from -80 to +120 mV in the control external solution (control, left panels) and after application of 20 μM FSK plus 10 μM of Esc peptides or GEN (right panels). **d** Time course of currents elicited in null FRT cells during addition of FSK+ 10 μM Esc peptide or GEN at different potentials. **e** Current ratio at +100 mV after application of FSK+ GEN or FSK + Esc peptides as indicated. Current intensity was normalized to that measured under control basal conditions (before addition of compounds). Stimulation with GEN or Esc peptides did not cause any increase of current in cells not expressing CFTR, and therefore it was not necessary to use the $CFTR_{Inh}$-172. Data are expressed as mean ± SEM from n≥3 independent experiments

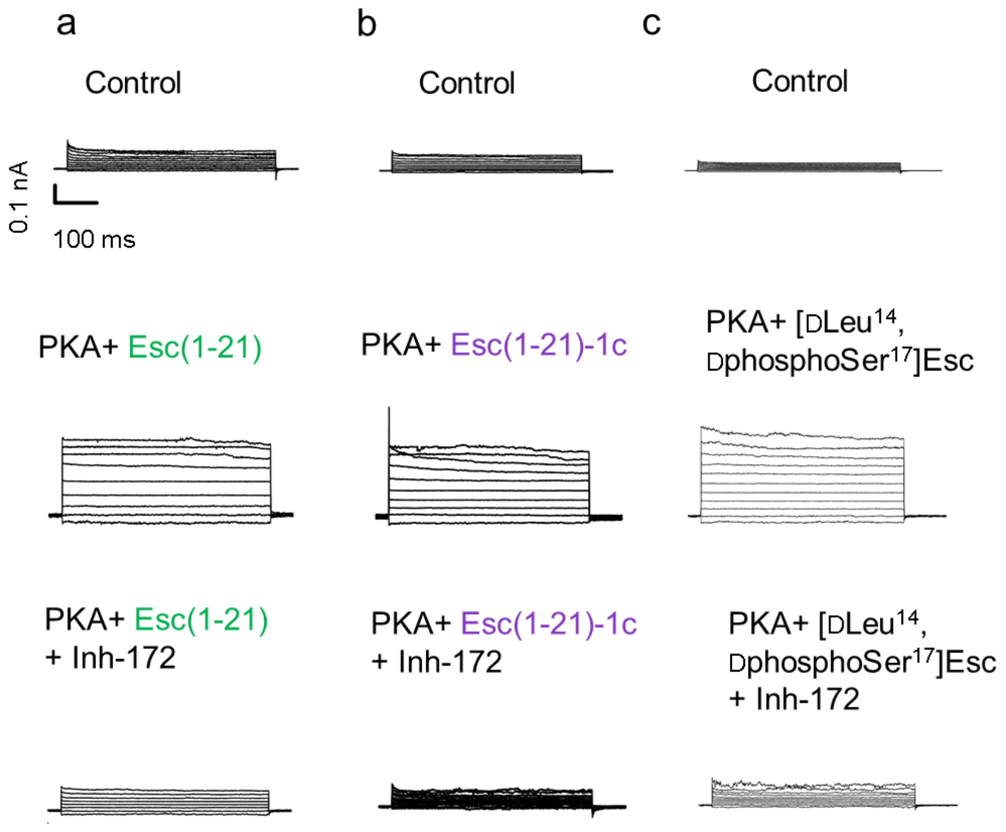

Fig. S4 Analysis of membrane patch in F508del-FRT cells from inside out patch technique. **a** Representative superimposed currents elicited at membrane potentials from -80 to +120 mV in the control solution (top panel); after addition of PKA+10 μM Esc(1-21) (central panel) and after subsesequent addition of 10 μM $CFTR_{Inh}$-172 (bottom panel). **b** The same as **a** but for 10 μM Esc(1-21)-1c. **c** The same as **a** but for 10 μM [DLeu^{14}, DphosphoSer^{17}]Esc

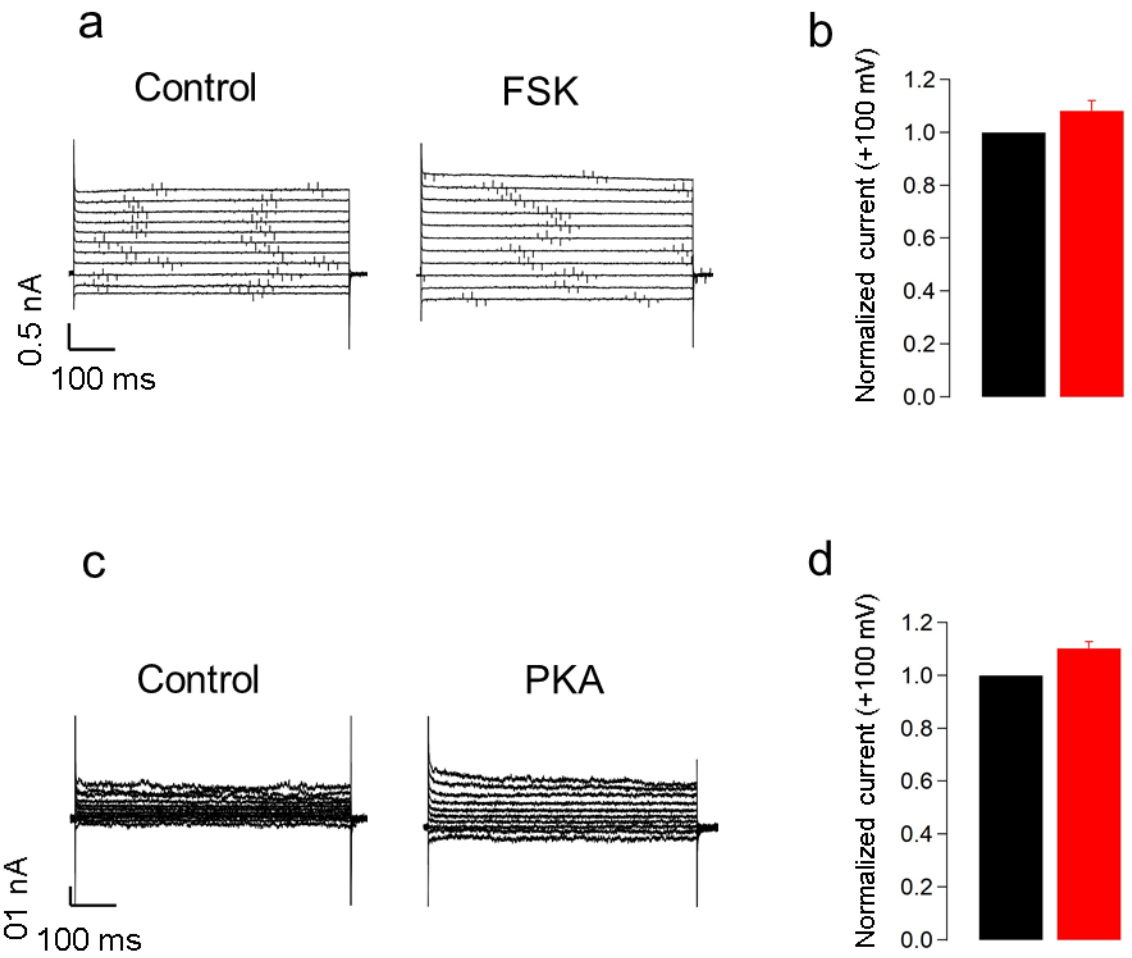

Fig. S5 Analysis of membrane currents in F508del-FRT cells from patch-clamp. **a** Representative superimposed currents elicited by whole-cell configuration at membrane potentials from -80 to +120 mV in the control solution (left panel) and after addition of 20 µM FSK (right panel). **b** Current ratio at +100 mV for indicated treatment (control basal condition, black bar; after CFTR activation by FSK, red bar). Current intensity was normalized to that measured under control basal conditions. Data are expressed as mean ± SEM from n≥3 independent experiments. **c** Representative superimposed currents elicited by inside-out configuration at membrane potential from -80 to +120 mV in the control solution (left panel) and after addition of 125 nM PKA (right panel). **d** Current ratio at +100 mV for indicated treatment (control basal condition, black bar; after CFTR activation by PKA, red bar). Current intensity was normalized to that measured under control basal conditions. Data are expressed as mean ± SEM from n≥3 independent experiments

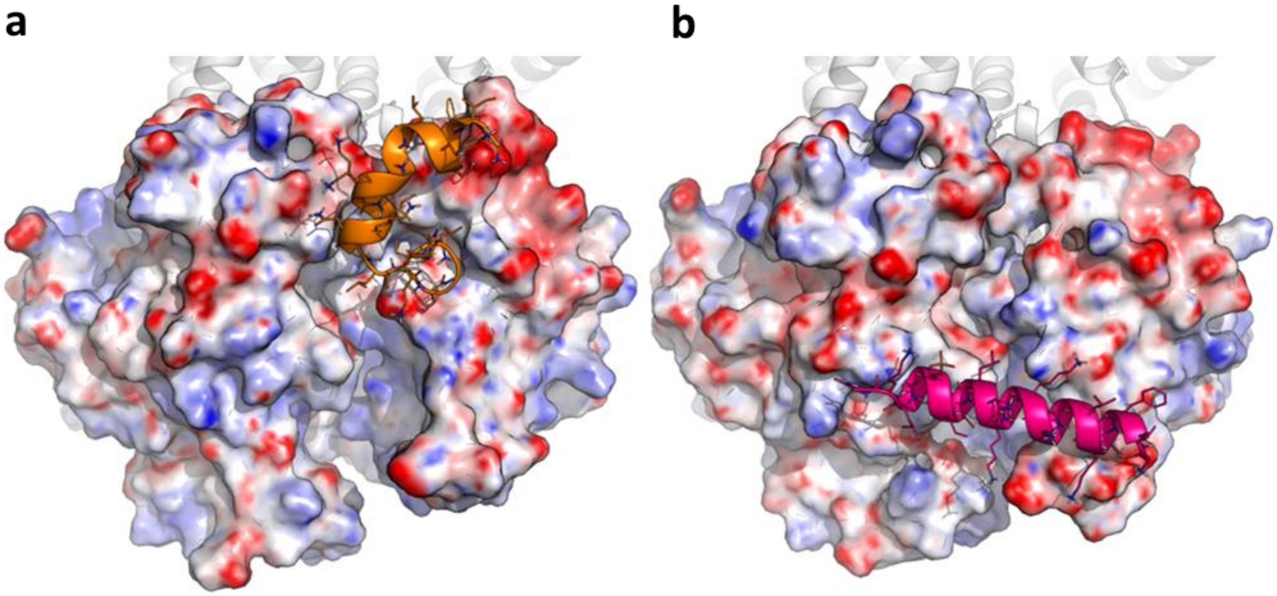

Fig. S6 Electrostatic surface potential of F508del NBDs in complex with peptides. **a** NBDs/Esc(1-21); **b** NBDs[DLeu^{14}DphosphoSer^{17}]Esc. The F508del NBDs are colored according with their electrostatic surface potential (red = negative charge; white = lipophilic/neutral; blue = positive charge); Esc(1-21) and [DLeu^{14}DphosphoSer^{17}]Esc are shown as cartoons and lines and are colored orange and hot pink, respectively
